# Utility and Cutoff Value of Hair Nicotine as a Biomarker of Long-Term Tobacco Smoke Exposure, Compared to Salivary Cotinine

**DOI:** 10.3390/ijerph110808368

**Published:** 2014-08-15

**Authors:** Sungroul Kim, Benjamin J. Apelberg, Erika Avila-Tang, Lisa Hepp, Dongmin Yun, Jonathan M. Samet, Patrick N. Breysse

**Affiliations:** 1 Department of Environmental Health Sciences, SoonChunHyang University, Asan 336-745, South Korea; E-Mail: balentain19@gmail.com; 2 Department of Epidemiology, Johns Hopkins Bloomberg School of Public Health, Baltimore, MD 21205, USA; E-Mails: bapelber@jhsph.edu (B.J.A.); etang@jhsph.edu; 3 Department of Health, Behavior and Society, Johns Hopkins Bloomberg School of Public Health, Baltimore, MD 21205, USA; E-Mail: hepplm@gmail.com; 4 Department of Preventive Medicine, University of Southern California, Los Angeles, CA 90089, USA; E-Mail: jsamet@med.usc.edu; 5 Department of Environmental Sciences, Johns Hopkins Bloomberg School of Public Health, Baltimore, MD 21205, USA; E-Mail: pbreysse@jhsph.edu

**Keywords:** biomarker, hair nicotine, salivary cotinine, cutoff value

## Abstract

While hair samples are easier to collect and less expensive to store and transport than biological fluids, and hair nicotine characterizes tobacco exposure over a longer time period than blood or urine cotinine, information on its utility, compared with salivary cotinine, is still limited. We conducted a cross-sectional study with 289 participants (107 active smokers, 105 passive smokers with self-reported secondhand smoke (SHS) exposure, and 77 non-smokers with no SHS exposure) in Baltimore (Maryland, USA). A subset of the study participants (n = 52) were followed longitudinally over a two-month interval. Median baseline hair nicotine concentrations for active, passive and non-smokers were 16.2, 0.36, and 0.23 ng/mg, respectively, while those for salivary cotinine were 181.0, 0.27, and 0.27 ng/mL, respectively. Hair nicotine concentrations for 10% of passive or non-smokers were higher than the 25th percentile value for active smokers while all corresponding salivary cotinine concentrations for them were lower than the value for active smokers. This study showed that hair nicotine concentration values could be used to distinguish active or heavy passive adult smokers from non-SHS exposed non-smokers. Our results indicate that hair nicotine is a useful biomarker for the assessment of long-term exposure to tobacco smoke.

## 1. Introduction

Assessment of the prevalence of tobacco use and of secondhand smoke (SHS) exposure is needed to estimate smoking-attributable disease burden and to evaluate the effectiveness of tobacco control programs throughout the world. Surveys assessing smoking with self-report have been commonly used to assess the prevalence and intensity of tobacco use and SHS exposure. However, due to increasingly overwhelming evidence of the harmful effect of tobacco use and changing social norms, under-reporting of tobacco use by active smokers has been documented [1]. Biomarkers of tobacco smoke exposure have been used to validate self-reporting, offering an objective measurement against which to compare self-reports.

Cotinine, a nicotine metabolite, is the most widely used biomarker [2,3]. It can be measured with great sensitivity in blood, saliva, or urine [2,4,5]. High correlations have been reported between saliva and serum cotinine concentrations (r = 0.8 or higher), with a saliva to serum ratio of 1.1–1.4 [6,7], and between urine and serum cotinine concentrations (r = 0.81), with a urine to serum ratio of five [6]. Cotinine in biofluids has been used as a marker for the amount of nicotine absorbed [4] and tobacco smoke exposure [8].However, cotinine has a relatively brief half-life of about 16–20 h and collection of liquid biosamples may reduce participant cooperation. Also, the handling and storage of samples can be challenging and costly in large studies.

During the past decade, hair nicotine concentration has increasingly been used as an alternative biomarker, because hair samples are easier to collect and less expensive to store and transport than biological fluids [9]. Moreover, hair nicotine, advantageously, characterizes tobacco exposure over a longer time period than blood or urine cotinine, with each cm length of hair representing approximately one month of exposure [10].

Previous research has shown that hair nicotine concentrations can be used to assess smoking status and SHS exposure among young children [11,12,13]. However, studies comparing concentrations of hair nicotine with other biomarkers to distinguish active smokers from nonsmokers among adult population are still limited. Additionally, cutoff values of hair nicotine for discriminating active smokers from nonsmokers have not been proposed. Such a cutoff value is necessary for the classification of smoking status and for evaluating misclassification rates in prevalence surveys and etiologic investigations. Furthermore, few studies have assessed smoking-related biomarker concentrations over time. Our aims in this study were to evaluate the utility of hair nicotine as biomarker of longer-term tobacco smoke exposure, to make a longitudinal comparison of hair nicotine and salivary cotinine, and to establish cutoff values that can be used to classify smoking status of adult population.

## 2. Experimental Section

### 2.1. Population

A total of 296 people aged 18 years or older of both sexes were recruited with a convenience sample approach from the Baltimore metropolitan area. We recruited the study population so as to ensure sufficient racial diversity but generalizability may be limited by relying on volunteers. The participants were recruited through advertisements in local newspapers and outside various commercial establishments, such as grocery stores and markets. The study population was categorized into three groups, based on their self-reported smoking status, including 77 non-smokers (NS) with limited exposure to SHS, 105 passive smokers (PS) with self-reported SHS exposure (*i.e.*, non-smokers who live with a smoker or are exposed to SHS in the workplace, such as bar and restaurant workers), and 107 active smokers (AS). We excluded pregnant women, people using nicotine replacement therapy, and smokeless tobacco users. Among the 296 participants, 34 of the PS group and 18 of the AS group provided follow-up samples (n = 52) after two months. Our study protocols were approved by the Johns Hopkins Institutional Review Board and we received informed consent from all individuals who agreed to participate in the study.

### 2.2. Questionnaire

Participants provided demographic information, including age, sex, education level, and race/ethnicity, as well as information on smoking behavior. We obtained information on daily and non-daily use of a variety of smoking products in the household, opportunities for in-home and at-work SHS exposure, smoking policies at home and in the workplace, and SHS exposure in other public places visited over the previous month.

### 2.3. Measurement of Hair Nicotine

Hair samples (approximately 30–50 strands) from each participant were taken from the back of the scalp where the growth pattern is the most uniform. In the laboratory, hair samples were trimmed to exclude hair that was more than 3 cm from the root end, representing the most recent 3-months of hair growth. The three cm-long samples (approximately 30 mg) were washed using 3 mL of dichloromethane and sonicated (Model 250HT, Aquasonic, Hayward, CA, USA) for 30 min to remove any nicotine adhered to the surface of the hair prior to nicotine extraction and analysis, as we were only interested in measuring nicotine accumulated by inhalation, systemic transport, and subsequent incorporation into the growing hair. Nicotine was extracted from the hair samples using an isotope dilution method with an internal standard (nicotine-d_3_, Supelco, St. Louis, MO, USA) [14]. Hair nicotine analysis was performed using gas chromatography/mass spectrometry (GC-17/MS-QP5000, Shimadzu, Canby, OR, USA) in selected ion monitoring (SIM) and splitless modes. For quality control, approximately 7% of the hair samples were subjected to duplicate analyses. The limit of detection (LOD) was 0.05 ng/mg for a 30-mg hair sample. The median recoveries from the nicotine-spiked hair samples within batches were 84% and 88% for the two concentration levels (0.67 and 3.3 ng/mg equal to 0.57 and 2.8 ng on column), respectively. The precision values ranged from 7% for 3.3-ng/mg nicotine-spiked hair samples within-batch to 20% for 0.67 ng/mg between the batches. A more complete description of the analytical method can be found elsewhere [14].

### 2.4. Measurement of Salivary Cotinine

To obtain saliva samples (2 mL, or as much as possible), participants were asked to spit into a test tube. Samples were stored in a cooler with ice and then transported to the laboratory at the Johns Hopkins Bloomberg School of Public Health. We extracted cotinine using a procedure that was modified slightly from that used for hair nicotine analysis; a total of 0.5 mL of saliva was mixed and equilibrated with cotinine-d_3_ as an internal standard using a horizontal shaker (KS 260 Basic, IKA, Wilmington, NC, USA) for one hour, then further processed as previously described [15]. Salivary cotinine concentrations were measured using GC-MS/MS (TSQ, Thermo, Waltham, MA, USA) coupled with an Rxi-5ms capillary column. The initial temperature for the capillary column was 50 °C (for 1 min), which was sequentially increased to 290 °C at a rate of 25 °C/min. The method detection limit (MDL) was 0.05 ng/mL at a 0.05-a.m.u scan width. The recovery was 90% for the standard solution of 0.64 ng/mL, and the relative standard deviation was 4% based on 8 repeated measurements of the standard solution (0.64 ng/mL).

To validate whether the differences in instrumental analytical procedures could have affected the comparisons between hair nicotine concentrations analyzed by using GC-MS and salivary cotinine concentrations analyzed by using GC-MS/MS for the same saliva samples (n = 82), we evaluated the relationship between the GC-MS and GC-MS/MS results (Supplementary Figure S1). Because cotinine concentrations are usually low in oral fluid, the MS/MS method is relevant when saliva is used [16].

### 2.5. Statistical Analysis

Pearson correlation coefficients and statistical significance of the correlations were calculated using SAS, version 9 (SAS Institute Inc., Cary, NC, USA). Data were log transformed when appropriate. The difference of mean log-transformed biomarker concentrations between baseline and follow-up time points was tested using paired t-test. Additionally, the association between log-transformed biomarker concentration and the self-reported number of cigarettes smoked was assessed using the Pearson correlation test and a multivariate regression model after controlling for other explanatory variables, such as age, gender, race, education level and hair treatment (for hair nicotine only). Cutoff values for hair nicotine and salivary cotinine concentrations were determined by Receiver Operating Characteristics (ROC) analysis [17].

## 3. Results

### 3.1. Concentrations of Hair Nicotine and Salivary Cotinine

The self-reported demographic characteristics obtained from the 289 participants are summarized in Table 1. The median age (45 years) for the PS group was 5 and 9 years older than those of the AS and NS groups, respectively. More females were recruited into the PS group (72%) than to the other two groups (60.5% for non-smokers and 64.1% for active smokers). Most of the NS group members (>70%) were Asian or Caucasian, whereas most PS and AS group members were African American or Caucasian. The NS group had a higher education level than the other two groups. Median (inter-quartile range, IQR) baseline hair nicotine concentration for the AS group was 16.2 ng/mg (3.9–41.4), which was significantly higher (*p* < 0.001) than the values of the other groups: 0.36 ng/mg (0.17–3.03) for the PS group, and 0.23 ng/mg (0.08–0.44) for the NS group. Similarly, median (IQR) baseline salivary cotinine concentration for the AS group (181 ng/mL (75.3–291.3)) for the AS group was significantly higher (*p* < 0.001) than for the PS (0.27 ng/mL (0.03–0.79)) and NS groups (0.26 ng/mL (0.03–0.61)).

**Table 1:** Demographic characteristics and biomarker concentrations of study population by self-reported smoking status.

Demographic Characteristics		Non-Smokers (baseline n = 77)	Passive Smokers (baseline n = 105)	Active Smokers (baseline n = 107)
Age, median years (IQR)		36 (26–53)	45 (34–52)	40 (27–51)
Female, %		60.5	72.1	64.1
Race, %				
Asian		36.8	9.5	9.4
African-American		13.1	51.4	45.8
White		40.7	26.7	32.0
Other		9.4	12.4	12.8
University or higher degree completed, %		75	24	41
Number of cigarettes per day, median (IQR)		-	-	15 (10–20)
Hair treatment, %		38.2	45.7	42.1
**Biomarker Concentrations**		**Non-Smokers**	**Passive Smokers**	**Active Smokers**
Hair nicotine (ng/mg), median (IQR) (% < LOD)	Baseline	0.23 (0.08–0.44) (82)	0.36 (0.17–3.03) (52)	16.2 (4.0–40.6) (2.8)
	Follow-up	-	0.29 (0.20–3.30) (59)	16.4 (3.3–27.3) (1.6)
Salivary cotinine (ng/mL), median (IQR) (% < LOD)	Baseline	0.27 (0.04–0.61) (30)	0.27 (0.04–0.80) (26)	181.0 (76.3–290.2) (2.8)
	Follow-up	-	0.41(0.035–1.08) (27)	135.1 (62.2–228.6) (0)

### 3.2. Associations of Biomarker Concentrations at Baseline and Follow-Up

**Figure 1:**
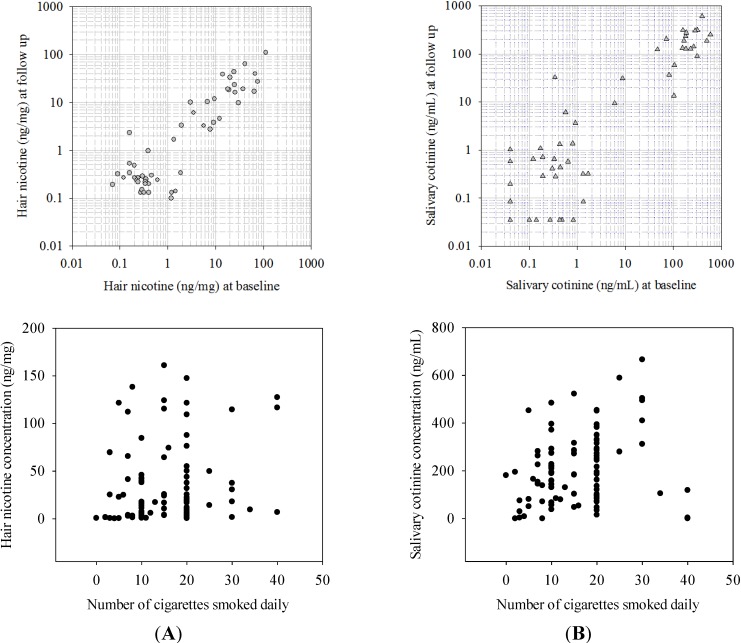
Associations of biomarker concentrations between baseline and follow-up and with the number of cigarettes smoked daily. (**A**) Hair nicotine; (**B**) Salivary cotinine.

The associations of biomarker concentrations at baseline with those measured at follow-up for the subset of AS and PS groups with follow-up samples (n = 52; 18 smokers and 34 passive smokers) are shown in Figure 1. Pearson correlation coefficients for log transformed biomarker concentrations at baseline and follow-up were similar for the two biomarkers: 0.88 (*p* < 0.001) for hair nicotine and 0.90 (*p* < 0.001) for salivary cotinine. Paired t-test results indicated that the mean concentration at baseline was not statistically significantly different from the mean concentration at follow-up (*t* = 0.71, *p* = 0.48 for hair nicotine, *t* = 0.15, *p* = 0.88 for salivary cotinine).

### 3.3. Association between Hair Nicotine, Salivary Cotinine and the Number of Cigarettes Smoked Daily

Among the self-reported AS group (n = 107), both hair nicotine and salivary cotinine concentrations showed a positive association with the number of cigarettes smoked daily (CSD) (r = 0.21, *p* = 0.04 and r = 0.41, *p* = 0.001 with 1 to 30 CSD; r = 0.24, *p* = 0.01 and r = 0.07, *p* = 0.46 with 1 to 40 CSD for hair nicotine and salivary cotinine, respectively) (Figure 1). In this study, approximately 28% of the variation of log-transformed hair nicotine concentrations was explained by the self-reported number of CSD and other explanatory variables, including age, gender, race, education level and hair treatment while 13% of the variation of salivary cotinine was explained in the multiple regression models (Table 2).

Furthermore, our model showed that hair nicotine and salivary cotinine concentrations increased by 5% (*p* = 0.003) and 3% (*p* = 0.02), respectively, with each 5-cigarette increase in daily smoking (5, 10, 15, 20, *etc.*) within the range from 1 to 40 cigarettes per day after adjusting for those explanatory variables among smokers (Table 2). Although it was not statistically significant (*p* = 0.11), hair nicotine concentrations for participants who had hair treatment were 62% of the values for those who have untreated hair, after controlling for the number of cigarettes smoked per day, age, gender, race and education level.

**Table 2:** Geometric means (GMs) and geometric mean ratios (GMRs) of hair nicotine and salivary cotinine concentrations per 5 cigarettes for self-reported smokers’ after adjustment for age, gender, race, education, and hair treatment.

Variable	Hair nicotine (Adjusted R^2^ = 0.28)			Salivary cotinine ( Adjusted R^2^ = 0.13)		
	Conc. (ng/mg)	*p*-value	Ratio (95% CI)	Conc. (ng/mL)	*p*-value	Ratio (95 % CI)
Number of cigarette smoked per day						
Per 5 cigarette	14.43	0.003	1.05 (1.02–1.09)	59.6	0.02	1.03 (1.01–1.05)
Age						
Per unit increase (age-median)	13.88	0.24	1.01 (0.99–1.04)	58.7	0.04	1.01 (1.00–1.03)
Gender (Reference = Male)						
Female	8.17	0.10	0.60 (0.32–1.12)	92.5	0.01	1.60 (1.12–2.27)
Race (Reference = White)						
Black	56.19	<0.0001	4.10 (2.28–7.38)	47.8	0.25	0.83 (0.59–1.15)
Education						
Per unit increase (low to high)	11.59	0.31	0.85 (0.61–1.17)	62.3	0.42	1.08 (0.90–1.29)
Hair treatment (Reference = No)						
Yes	8.45	0.11	0.62 (0.34–1.11)	Not applicable		

### 3.4. Comparison of Biomarker Levels for Nonsmokers and Passive Smokers

**Figure 2:**
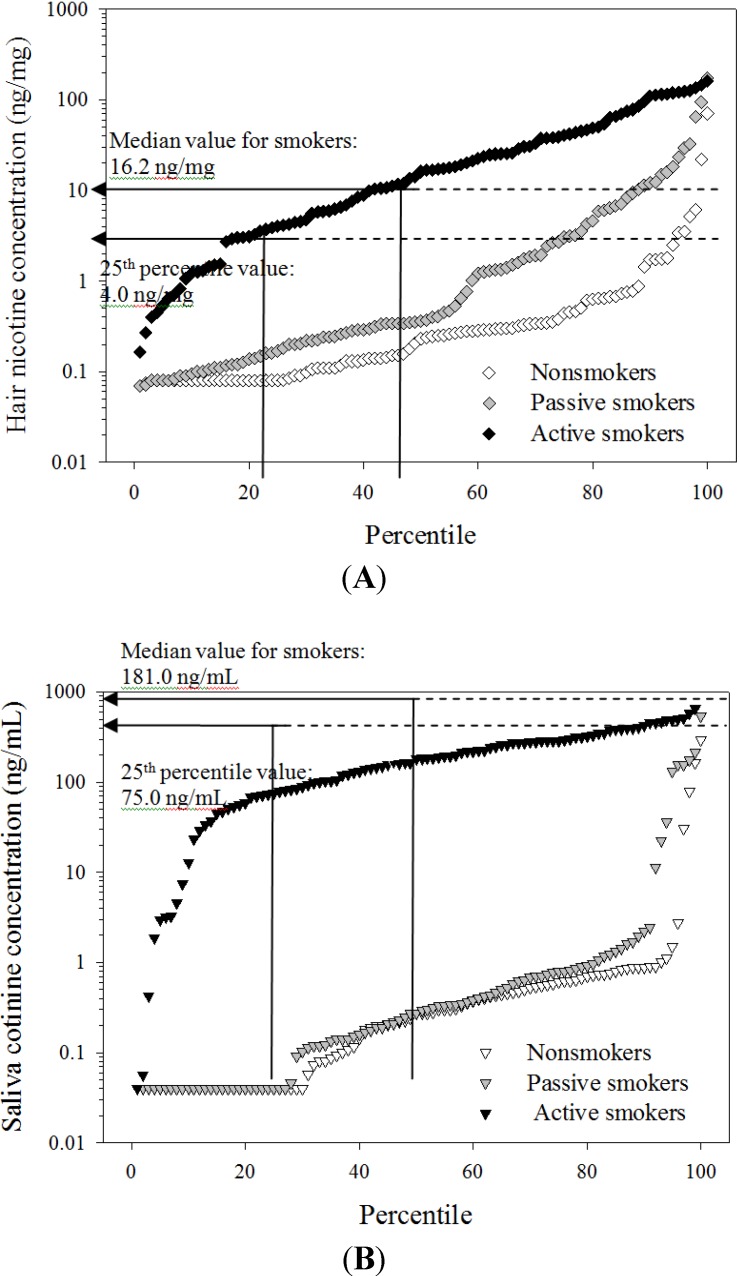
Distributions of hair nicotine and salivary cotinine concentrations by self-reported smoking status and 25th and 50th percentile values obtained from self-reported active smokers. Three different shades (black, gray, and white) represent for active smokers, passive smokers and nonsmokers, separately.) (**A**) Hair nicotine; (**B**) Salivary cotinine.

As seen in the Figure 2, we observed that the median concentration of hair nicotine of PS was higher than that of NS whereas, in the case of salivary cotinine, the median values were comparable for the PS and NS groups.

Also, Figure 2 shows two sets of lines representing the 25th and 50th percentile values obtained from the AS group for both the salivary cotinine and the hair nicotine concentrations. As expected, biomarker levels for most NS and PS group members were lower than the 25th percentile of the AS group. However, among the hair and saliva biomarker concentration pairs, nineteen of the PS (n = 16) and NS (n = 3) samples were higher than the 25th percentile value for hair nicotine in the AS group (4.0 ng/mg); by contrast, salivary cotinine concentrations of all corresponding individuals were lower than the 25th percentile value (75 ng/mL) (Supplementary Figure S2).

### 3.5. Cutoff Values of Hair Nicotine and Salivary Cotinine

**Figure 3:**
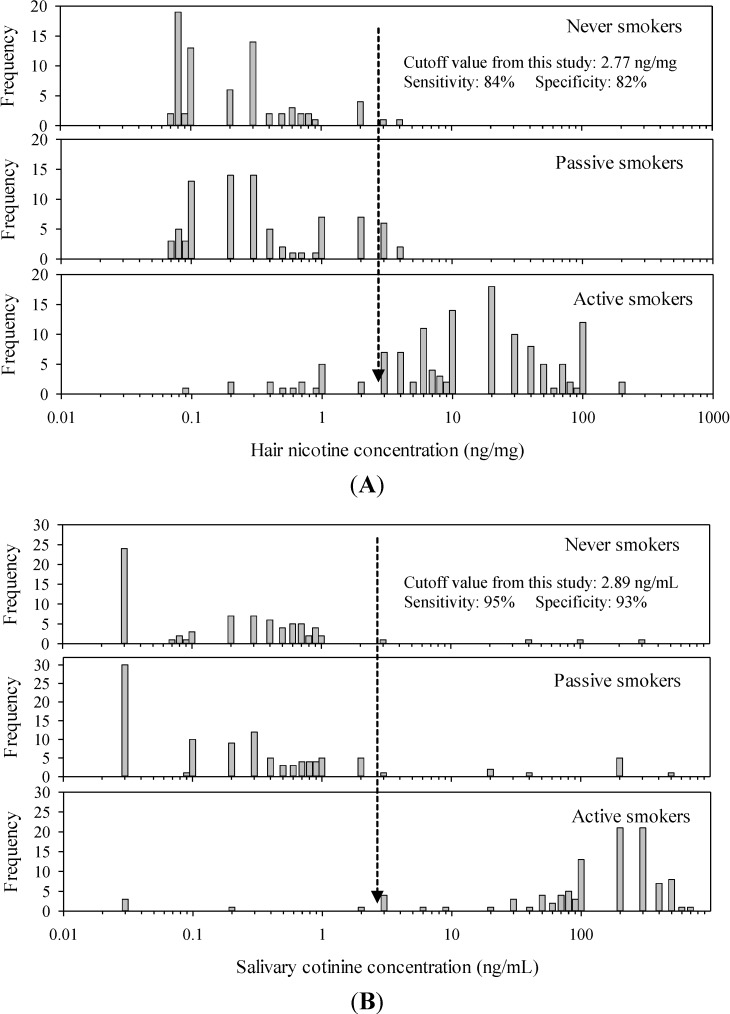
Frequency of (**A**) hair nicotine and (**B**) salivary cotinine concentration(s) by self-reported smoking status and cutoff values distinguishing smokers from nonsmokers and their sensitivities and specificities obtained from ROC analyses.

The performances of the two biomarkers in distinguishing AS from NS and PS groups were evaluated using ROC curves. Supplementary Table 1 summarizes the sensitivities and specificities at various cutoff values. Cutoff values were establishede to distinguish AS from PS and NS group members. We selected 2.77 ng/mg and 2.89 ng/mL, which exhibited the greatest degree of correct classification rate, as a cutoff value for hair nicotine and salivary cotinine, respectively. For hair nicotine, the sensitivity and the specificity of the selected cutoff value were 84% and 82%, respectively; for salivary cotinine, these values were 95% and 93%, respectively (Figure 3).

## 4. Discussion

Our findings indicate that hair nicotine is a useful biomarker for the assessment of long-term tobacco smoke exposure. Our study also provides cutoff value of hair nicotine as well as salivary cotinine for distinguishing AS from PS and NS.

### 4.1. Correlation between Hair Nicotine and Salivary Cotinine Concentrations

Our study showed a weak correlation between the two biomarkers. A possible explanation for the low correlation between hair nicotine and salivary cotinine concentrations is the different molecular kinetics of the two biomarkers. As the half-life of cotinine is 16–20 h [18], salivary cotinine is more readily affected by day-to-day variation in tobacco or SHS smoke exposure; indicating that cotinine concentration may better reflect short-term exposure, *i.e.*, that immediately prior to sample collection (hours or days). By contrast, the hair nicotine concentration in the present study represents cumulative exposure over a 3-month period and provides an indication of regular and long-term tobacco smoke exposure.

### 4.2. Association of Hair Nicotine with the Number of Cigarette Smoked Daily

Hair nicotine concentration has been used as a long-term biomarker of tobacco smoke exposure [11,14,19,20,21,22,23,24]. With hair growth reported to be approximately 1 cm/month [10], a small amount of hair (2–3 cm) from the scalp can potentially represent exposure to tobacco smoke over a period of 2–3 months.

In this study, hair nicotine concentrations were more consistent between the baseline and 2-month follow-up time points among the self-reported active smokers (r = 0.88, *p* = 0.001 for hair nicotine and r = 0.70 *p* = 0.006 for salivary cotinine). However, hair nicotine exhibited lower correlation (*r* = 0.21, *p* = 0.04) with the self-reported number of CSD (ranging from 1 to 30) among the smokers than salivary cotinine (*r* = 0.41, *p* = 0.001). Because hair nicotine is a longer-term biomarker, it is not surprising that it is not as strongly correlated with self-reported recent smoking behavior (*i.e.*, cigarettes smoked per day) as salivary cotinine, which represents relatively recent exposure levels (within approximately 2 days).

A similarly weak association between the number of cigarettes smoked daily with biomarkers (exhaled carbon monoxide and plasma cotinine) has recently been reported by Ho *et al.* in a study (*r* = 0.32–0.39, *p* < 0.001) conducted with African American light smokers (*n* = 700) [25]. Joseph *et al.* also suggested that the nonlinear relationship between biomarkers and the number of CSD may be due to a plateau at higher levels of smoking [26]. Smokers can extract varying levels of nicotine by altering their smoking topography (e.g., puff volume, number of puffs) even though they reported the same number of cigarettes smoked per day, which in turn can affect the level of internal dose of nicotine absorbed and result in reducing the association of biomarkers with the number of cigarettes smoked [27,28].

### 4.3. Hair Nicotine as a Biomarker of SHS Exposure

Although, limited studies have been conducted with adult population to examine the utility of hair nicotine as a biomarker of SHS exposure, a few studies have been conducted with study populations of young children. Nafstad *et al.* compared three methods for estimating SHS exposure among children aged 12–36 months. They reported that the correlation coefficient between hair nicotine and CSD was 0.64 whereas that between the urinary cotinine to creatinine ratio and CSD was 0.50 [3]. Furthermore, their study concluded that hair nicotine concentration might better distinguish non-SHS exposed children from SHS-exposed children. Al-Delaimy *et al.* also reported similar results [11]. They measured 297 hair and 158 urine samples collected from children aged 3–27 months and found that children’s hair nicotine concentrations had a stronger association (Chi^2^ = 142.1) with household smoking habits (no smokers, smoke only outside, smoke inside) than urine cotinine concentrations (Chi^2^ = 49.5). They also reported that the number of smokers in the household, and the number of cigarettes smoked by parents and other members of the household exhibited stronger associations with hair nicotine (r^2^ = 0.55) than urinary cotinine concentration (r^2^ = 0.31) in their multivariate regression modeling results, after adjusting for other explanatory variables. Although our study population consisted of adults, our finding of the greater validity of hair nicotine for distinguishing heavy passive smokers from non-smokers was consistent with the results of these previous studies conducted in children populations.

We examined a possible source of nicotine exposure in those 19 NS and PS group members whose hair nicotine concentrations were higher than the 25th percentile values of that of the AS group, while corresponding salivary cotinine concentrations were lower than the comparative 25th percentile values. As mentioned in the Experimental Section, because we excluded people on nicotine replacement therapy, smokeless tobacco users, and pregnant women from our study population before we started the study, it is unlikely that such high values were affected by the use of nicotine patches. Second, it has been reported that tomatoes, potatoes, cauliflower, and green peppers contain small amounts of nicotine. However, those 19 NS and PS members’ biomarker levels were similar to active smokers’ 25th percentile levels. Therefore, with consideration of half-lives of nicotine (2–3 h) and cotinine (17–19 h) in human body, we believe it is impossible to eat sufficient amounts of food to have biomarker levels equal to those of active smokers. Finally, using the questionnaire data, we conducted a further examination of the SHS exposure status of those 19 NS and PS group members who had hair nicotine concentrations which are higher than 25th percentile of that of the AS group and salivary cotinine concentrations lower than the comparative value. Sixteen of the 19 individuals had self-reported SHS exposure and were living with regular smokers in homes without a smoking policy. These individuals were likely to have been exposed to high levels of SHS in the long-term as indicated by their elevated hair nicotine concentrations. The lower salivary cotinine concentration suggests that their recent exposure was low. These results imply that hair nicotine concentration may be a useful predictor of mean SHS exposure status. However, to ensure an accurate evaluation, further study may be warranted.

### 4.4. Cutoff Values

In this study, we used two biomarkers to validate self-reported smoking status. We determined cutoff values for classifying a person as a smoker or non-smoker with sufficient sensitivity and specificity: 2.77 ng/mg for hair nicotine and 2.89 ng/mL for salivary cotinine.

The sensitivity and specificity for the corresponding cutoff values were 84% and 82%, respectively, for hair nicotine and 95% and 93%, respectively, for salivary cotinine. Jarvis *et al.* have reported that the optimal cutoffs for salivary nicotine, cotinine, and thiocyanate measures [29]. High sensitivity is necessary to minimize misclassification of true smokers as nonsmokers [30]. The sensitivity and specificity values from our hair nicotine study were similar to those (78% sensitivity and 84% specificity) for hair cotinine cutoff values obtained from non-pregnant women in a different study (0.8 ng/mg) [30]. Our salivary cotinine cutoff value, however, was similar to recent findings (3.0 ng/mg) reported by Benowitz *et al.* [31] who determined the serum cotinine cutoff value in the U.S. population using data from the National Health and Nutrition Examination Survey **(**NHANES) between 1988 and 2004.

In general, non-smokers are not expected to report themselves as smokers, but owing to exposure to SHS, it is possible for them to have a positive biomarker concentration, which reduces specificity to less than 100%. Internal dose levels of SHS cumulative exposure measured by hair nicotine analysis may not be consistent with participants’ recall of recent exposures events, and may further reduce the specificity to more than that of salivary cotinine, which reflects relatively recent exposure. Despite the slightly lower specificity of hair nicotine, the detection of high nicotine levels from hair samples of heavy passive smokers among our study population may have an important impact because it suggests a potential to under-ascertain SHS exposure using cotinine. Underreporting can lead to underestimating the potential association between SHS exposure and disease risk [32]. Thus, application of a longer term biomarker such as hair nicotine may be relevant for evaluating health outcomes related to SHS exposure.

Our study had several limitations. Our results and cutoff values should be interpreted with care because the values were obtained from a relatively small number of samples (a total of 289). Although recruitment was conducted in various ways, including advertisements in local newspapers, and in person, outside various commercial establishments, such as grocery stores and markets; our sample might not be representative. Given the small number of samples, the cutoff values we generate should be interpreted with caution as they are unlikely to be representative of the general population.

Furthermore, we had only 52 subjects who agreed to participate in the follow-up study by providing their hair and saliva samples 2 months after the baseline examination. A future study with a larger sample size may be needed to address interindividual variability within and between biomarkers. Due to the complexity and expense of the study, a larger population-based representative sample, was not considered feasible. Consequently, we could not evaluate the effect of race, age, sex and other potential determinants on biomarker concentrations. Also, our analyses assume that hair growth rate and nicotine metabolism rate are similar across different racial/ethnic groups.

## 5. Conclusions

Despite the study limitations listed above, our study presented optimal cutoff value of hair nicotine and its utility, compared to salivary cotinine using adult population-based data. Salivary cotinine is a well-established and excellent biomarker of recent tobacco exposure. Our results showed that the hair nicotine values could be used to distinguish active or heavy passive smokers from non-SHS exposed nonsmokers. Hair nicotine is a useful biomarker for the assessment of long-term exposure to tobacco smoke. Researchers can select the appropriate biomarker for evaluating long-term or recent exposure.

## Supplementary Files

Supplementary File 1
