# Supplementary material for: Utility and Cutoff Value of Hair Nicotine as a Biomarker of Long-Term Tobacco Smoke Exposure, Compared to Salivary Cotinine
Source: Int J Environ Res Public Health. 2014 Aug 15;11(8):8368–82. doi: 10.3390/ijerph110808368 (PMC4143866; doi:10.3390/ijerph110808368)
Supplement: Supplementary File 1 [file ijerph-11-08368-s001.pdf]

Utility and Cutoff Value of Hair Nicotine as a Biomarker of Long-Term Tobacco Smoke Exposure, Compared to Salivary Cotinine

Figure S1. The relationship between the GC-MS and GC-MS/MS results for the same saliva samples randomly selected (the ratio of salivary cotinine concentrations from GC-MS/MS to GC-MS was 1.2 with the correlated coefficient of 0.91 for the saliva samples randomly selected (n = 82)).

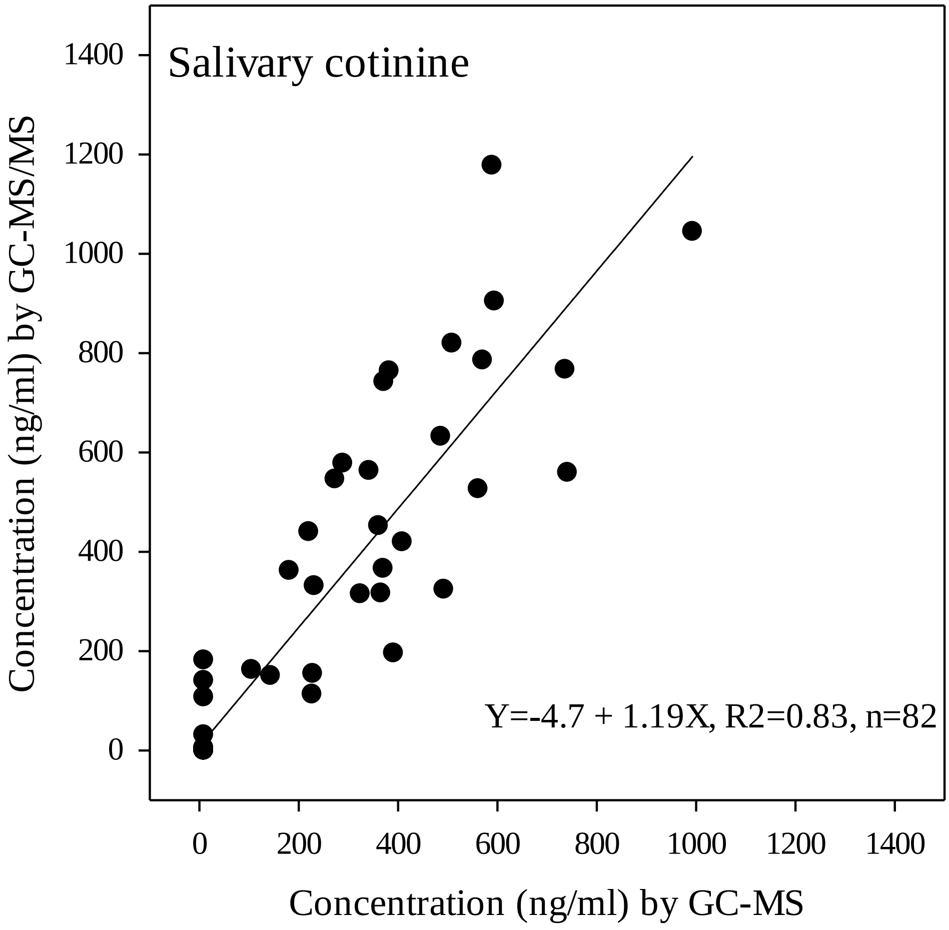

Table S1. Sensitivities and specificities for cutoff values of salivary cotinine and hair nicotine obtained before and after changing smoking status for seven individuals (from nonsmokers to smokers).

**Salivary cotinine (n = 289)**							
**Before changing smoking status**				**After changing smoking status**			
**Cutoff value**	**Sensitivity (%)**	**Specificity (%)**	**Correctly classified (%)**	**Cutoff value**	**Sensitivity (%)**	**Specificity (%)**	**Correctly classified (%)**
1.21	96.26	87.91	91.00	1.21	96.49	91.43	93.43
1.33	96.26	89.01	91.70	1.33	96.49	92.57	94.12
1.55	96.26	89.56	92.04	1.55	96.49	93.14	94.46
1.67	95.33	90.11	92.04	1.67	95.61	93.71	94.46
2.12	95.33	91.21	92.73	2.12	95.61	94.86	95.16
2.30	95.33	91.76	93.08	2.30	95.61	95.43	95.50
2.53	95.33	92.31	93.43	2.53	95.61	96.00	95.85
2.81	95.33	92.86	93.77	2.81	95.61	96.57	96.19
2.89	**95.33**	**93.41**	**94.12**	**2.89 ****	95.61	97.14	96.54
3.18	94.39	93.41	93.77	3.18	94.74	97.14	96.19
3.19	93.46	93.41	93.43	3.19	93.86	97.14	95.85
3.27	92.52	93.41	93.08	3.27	92.98	97.14	95.50
5.99	91.59	93.41	92.73	5.99	92.11	97.14	95.16
8.72	90.65	93.41	92.39	8.72	91.23	97.14	94.81
15.40	89.72	93.41	92.04	15.40	90.35	97.14	94.46
15.53	88.79	93.41	91.70	15.53	89.47	97.14	94.12
**Hair nicotine (n = 289)**							
**Before changing smoking status**				**After changing smoking status**			
**Cutoff value**	**Sensitivity (%)**	**Specificity (%)**	**Correctly classified (%)**	**Cutoff value**	**Sensitivity (%)**	**Specificity (%)**	**Correctly classified (%)**
1.75	84.11	77.47	79.93	1.75	85.09	80.57	82.35
1.81	84.11	78.02	80.28	1.81	85.09	81.14	82.70
1.87	84.11	79.12	80.97	1.87	85.09	82.29	83.39
1.94	84.11	80.22	81.66	1.94	85.09	83.43	84.08
2.50	84.11	80.77	82.01	2.5	85.09	84.00	84.43
2.66	84.11	81.32	82.35	2.66	85.09	84.57	84.78
2.69	84.11	81.87	82.70	2.69	85.09	85.14	85.12
2.77	**84.11**	**82.42**	**83.04**	**2.77 ****	85.09	85.71	85.47
2.91	83.18	82.42	82.70	2.91	84.21	85.71	85.12
3.00	82.24	82.42	82.35	3.00	83.33	85.71	84.78
3.01	81.31	82.42	82.01	3.01	82.46	85.71	84.43
3.03	80.37	82.42	81.66	3.03	81.58	85.71	84.08
3.06	80.37	82.97	82.01	3.06	81.58	86.29	84.43
3.11	79.44	82.97	81.66	3.11	80.70	86.29	84.08
3.17	79.44	83.52	82.01	3.17	80.70	86.86	84.43
3.37	78.50	84.07	82.01	3.37	79.82	87.43	84.43

Notes: ***** Cutoff values were obtained with those nonsmoker outliers (n = 7) who reported themselves as non-smokers but whose two biomarker levels (11~96 ng/mg for hair nicotine and 154~536 ng/mL for saliva cotinine) were close to or higher than the median values for active smokers (*i.e.*, 16.2 ng/mg for hair nicotine and 181 ng/mL). Cutoff values were same without regard to the existence of misclassification. ****** Bolded values exhibited the greatest degree of correct classification rate.

Figure S2. Hair nicotine and salivary cotinine concentrations among self-reported non-smokers and passive smokers and 25th and 50th percentile values obtained from self-reported active smokers. (Those 7 (black dots) participants had hair nicotine and saliva cotinine concentrations which were close to or higher than the median values of self-reported smokers. Those 19 (grey dots) NS or PS participants had hair nicotine concentrations that were higher than the 25th percentile value for that in the AS group, by contrast, saliva cotinine concentrations of all corresponding individuals were lower than the 25th percentile value in the AS group).

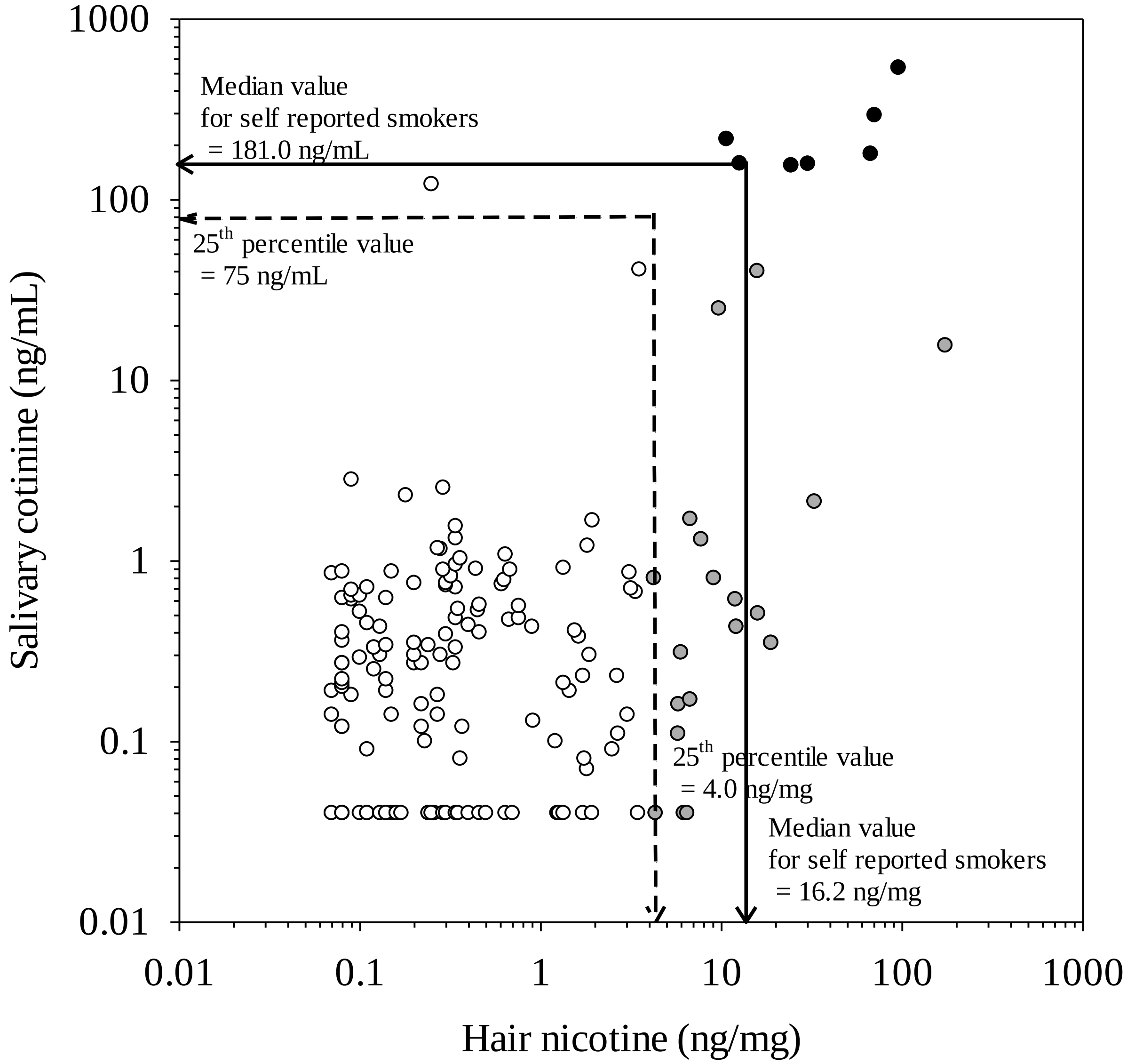

© 2014 by the authors; licensee MDPI, Basel, Switzerland. This article is an open access article distributed under the terms and conditions of the Creative Commons Attribution license (http://creativecommons.org/licenses/by/3.0/).
